# Diagnostic Performance and Clinical Impact of ^68^Ga-PSMA-11 PET/CT Imaging in Early Relapsed Prostate Cancer After Radical Therapy: A Prospective Multicenter Study (IAEA-PSMA Study)

**DOI:** 10.2967/jnumed.120.261886

**Published:** 2022-02

**Authors:** Juliano J. Cerci, Stefano Fanti, Enrique E. Lobato, Jolanta Kunikowska, Omar Alonso, Sevastian Medina, Fuad Novruzov, Thabo Lengana, Carlos Granados, Rakesh Kumar, Venkatesh Rangarajan, Akram Al-Ibraheem, Mukbil Hourani, Nor S. Ali, Azra Ahmad, Zohar Keidar, Ozlem Küçük, Umut Elboga, Mateos Bogoni, Diana Paez

**Affiliations:** 1 Quanta Diagnóstico e Terapia, Curitiba, Brazil;; 2 IRCCS Azienda Ospedaliero-Universitaria di Bologna, Bologna, Italy;; 3 Division of Human Health, International Atomic Energy Agency, Vienna, Austria;; 4 Nuclear Medicine Department, Medical University of Warsaw, Warsaw, Poland;; 5 Centro Uruguayo de Imagenología Molecular (CUDIM), Montevideo, Uruguay;; 6 Instituto Nacional de Cancerologia, Tlalpan, Mexico;; 7 Nuclear Medicine Department, National Centre of Oncology, Baku, Azerbaijan;; 8 University of Pretoria, Pretoria, South Africa;; 9 Instituto Nacional de Cancerologia, Bogotá, Colombia;; 10 All India Institute of Medical Sciences, New Delhi, India;; 11 Tata Memorial Centre, Mumbai, India;; 12 King Hussein Cancer Center, Amman, Jordan;; 13 American University of Beirut Medical Center, Beirut, Lebanon;; 14 Institute Kanser Negara, Putrajaya, Malaysia;; 15 Pakistan Atomic Energy Commission (PAEC), Islamabad, Pakistan;; 16 Rambam Medical Centre, Haifa, Israel;; 17 Ankara University, Ankara, Turkey; and; 18 University of Gaziantep, Gaziantep, Turkey

**Keywords:** PSMA, PET/CT, prostate cancer, biochemical relapse

## Abstract

Biochemical recurrence (BCR) is a clinical challenge in prostate cancer (PCa) patients, as recurrence localization guides subsequent therapies. The use of PET with prostate-specific membrane antigen (PSMA) provides better accuracy than conventional imaging practice. This prospective, multicenter, international study was performed to evaluate the diagnostic performance and clinical impact of PSMA PET/CT for evaluating BCR in PCa patients in a worldwide scenario. **Methods:** Patients were recruited from 17 centers in 15 countries. Inclusion criteria were histopathologically proven prostate adenocarcinoma, previous primary treatment, clinically established BCR, and negative conventional imaging (CT plus bone scintigraphy) and MRI results for patients with PSA levels of 4–10 ng/mL. All patients underwent PET/CT scanning with ^68^Ga-PSMA-11. Images and data were centrally reviewed. Multivariate logistic regression analysis was applied to identify the independent predictors of PSMA-positive results. Variables were selected for this regression model on the basis of significant associations in the univariate analysis and previous clinical knowledge: Gleason score, the PSA level at the time of the PET scan, PSA doubling time, and primary treatment strategy. All patients were monitored for a minimum of 6 mo. **Results:** From a total of 1,004 patients, 77.7% were treated initially with radical prostatectomy and 22.3% were treated with radiotherapy. Overall, 65.1% had positive PSMA PET/CT results. PSMA PET/CT positivity was correlated with the Gleason score, PSA level at the time of the PET scan, PSA doubling time, and radiotherapy as the primary treatment (*P* < 0.001). Treatment was modified on the basis of PSMA PET/CT results in 56.8% of patients. PSMA PET/CT positivity rates were consistent and not statistically different among countries with different incomes. **Conclusion:** This multicenter, international, prospective trial of PSMA PET/CT confirmed its capability for detecting local and metastatic recurrence in most PCa patients in the setting of BCR. PSMA PET/CT positivity was correlated with the Gleason score, PSA level at the time of the PET scan, PSA doubling time, and radiotherapy as the primary treatment. PSMA PET/CT results led to changes in therapeutic management in more than half of the cohort. The study demonstrated the reliability and worldwide feasibility of PSMA PET/CT in the workup of PCa patients with BCR.

## Visual Abstract

**Figure:**
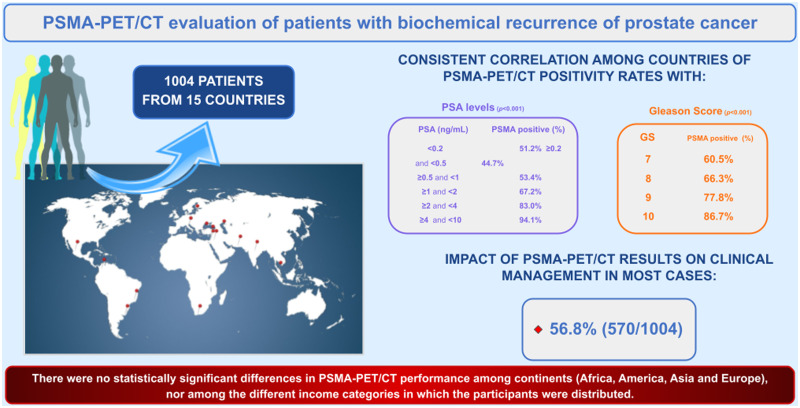

Prostate cancer (PCa) is the second most common cancer in men, accounting for 7.8% of all cancers in this population (*1*). Greater life expectancy worldwide and improved access to screening and diagnostic methods in developing nations are mainly responsible for the current trend of increasing incidence (*2*).

Initial treatment with curative intent is feasible, with radical prostatectomy or radiotherapy; nevertheless, early recurrence occurs in up to 50% of patients within 10 y (*3*–*5*). Biochemical recurrence (BCR) is defined as increasing serum prostate-specific antigen (PSA) levels after initial treatment, under specific criteria (*6*–*8*).

The key question for proper treatment planning in BCR remains whether the rise in PSA levels is reflective of local, regional, or distant recurrence. With increasing rates of success of early salvage therapy, the diagnosis of local tumor recurrence at the earliest possible stage has become pertinent. Salvage radiotherapy after radical prostatectomy has been shown to be most effective—reaching a durable response—when the postoperative PSA level is preferably below 0.5 ng/mL, with better outcomes when the PSA level is below 0.2 ng/mL (*4*,*9*).

Despite guidelines indicating that prostate-specific membrane antigen (PSMA) PET/CT is the imaging modality of choice in BCR (*10*–*17*), in some countries—especially those with lower incomes—conventional imaging with CT and bone scintigraphy are still being used, even if the diagnostic yield of these techniques is low, especially for patients with low PSA levels (*11*).

Most PSMA PET/CT studies have been performed at a single institution or were retrospectively planned. Furthermore, most reported studies have been conducted at academic centers in highly developed countries; thus, to our knowledge, there are no data from large prospective international trials. The International Atomic Energy Agency initiated a Coordinated Research Project to evaluate the feasibility and usefulness of PSMA PET/CT for studying PCa patients with BCR in 15 countries to inform international practice.

The primary aim of this prospective study was to evaluate the diagnostic performance of PSMA PET/CT in PCa patients with BCR worldwide, through an international multicenter effort, and the impact of PSMA PET/CT on clinical management.

## MATERIALS AND METHODS

### Study Design

Two investigators’ meetings were held, in 2017 and 2019. The first defined the study protocol, whereas in the second, an interim evaluation was performed, together with image and data review. The study followed a prospective, multicenter, international design, encompassing 17 centers in 15 countries (Azerbaijan, Brazil, Colombia, India, Israel, Italy, Jordan, Lebanon, Malaysia, Mexico, Pakistan, Poland, South Africa, Turkey, and Uruguay). Standard forms for data registration were developed and agreed on by the investigators. Data were collected for PSMA PET/CT positivity rate, localization of positive findings, and impact on patient management (Supplemental Fig. 1) (supplemental materials are available at http://jnm.snmjournals.org). All centers obtained local ethics clearance for prospective recruitment of patients and data collection, according to national regulations. All subjects signed an informed consent form.

### Patients

Patients who had histopathologically proven prostate adenocarcinoma, who had undergone primary definitive treatment (radical prostatectomy or radiotherapy), and who had BCR were recruited. All patients were monitored for a minimum of 6 mo after PSMA PET/CT.

The 6 inclusion criteria were an age of greater than 18 y; histopathologically proven prostatic adenocarcinoma; previous primary treatment for PCa (radical prostatectomy or radiotherapy); BCR, defined as a PSA level above 0.2 ng/mL, confirmed by 2 subsequent consecutive measurements, after radical prostatectomy, or as an absolute increase in the PSA level of 2 ng/mL above the nadir after radiotherapy; negative conventional imaging (CT plus bone scintigraphy) and MRI results for patients with PSA levels of 4–10 ng/mL; and written informed consent.

The 3 exclusion criteria were a history of any malignancy other than PCa; a history of Paget disease; and BCR and PSA levels of greater than or equal to 10 ng/mL.

### PET/CT Imaging

All patients underwent PSMA PET/CT with the same radiopharmaceutical, ^68^Ga-PSMA-11 (*18**–**21*), which was synthesized at the radiopharmaceutical laboratory of each participating center. PET studies were performed on dedicated PET/CT scanners, and image quality was evaluated by board-certified nuclear medicine physicians.

According to the methodology proposed in the medical literature (*10*), patients were administered ^68^Ga-PSMA-11 (2 MBq/kg; a minimum of 125 MBq) by slow intravenous injection. At 60 to 90 min after injection, standard image acquisition was performed. Low-dose (diagnostic) CT images were obtained from the midthigh to above the orbitomeatal line. Three-dimensional PET images were acquired for the same body extension, for at least 2 min/bed position. Real true-body images (images from head to toes), contrast-enhanced CT, and diuretic and late images were allowed.

PET/CT studies were assessed by 2 board-certified nuclear medicine physicians with extensive experience in PSMA PET/CT oncologic imaging at each center, and all scans were later centrally reviewed. Discordant findings were addressed at consensus meetings, and final results were used for analysis.

### PET/CT Image Analysis

**FIGURE 1.**
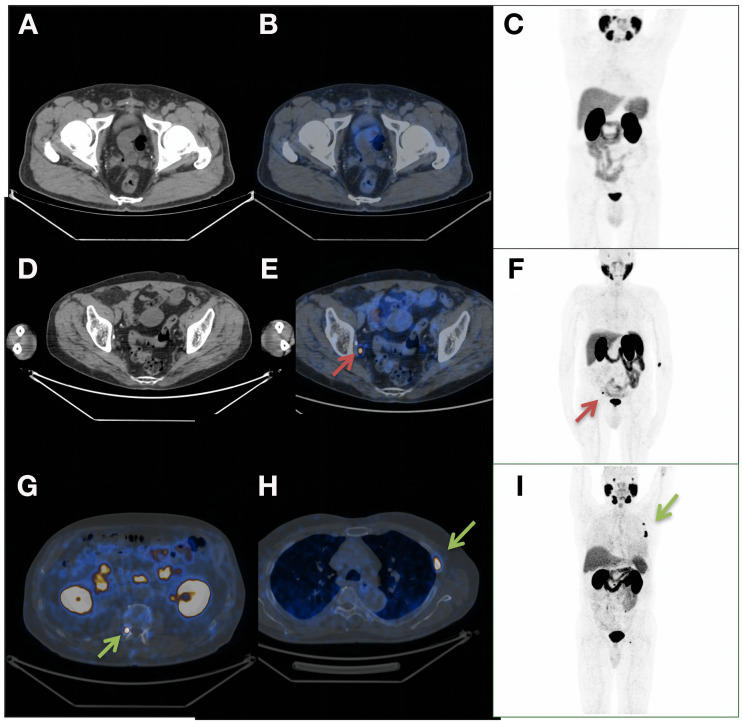
(A–C) Negative PSMA PET/CT results for 65-y-old patient who had undergone radical prostatectomy plus PNLD and had T3bN0 BCR (PSA, 0.55 ng/mL). Treatment plan was not altered by PSMA PET/CT results (radiotherapy) (A: axial CT; B: axial fusion; C: maximum-intensity projection [MIP]). (D–F) Positive PSMA PET/CT results for 67-y-old patient who had undergone radical prostatectomy plus PNLD and who had T2aN1 BCR (PSA, 0.4 ng/mL). Treatment plan was modified from radiotherapy to ADT (D: axial CT; E: axial fusion; F: MIP) for 0.4-cm lymph nodes (red arrows). (G–I) Positive PSMA PET/CT results for 65-y-old patient who had undergone radical prostatectomy plus PNLD and who had T3aN0 BCR (PSA, 0.2 ng/mL). Treatment plan was modified from radiotherapy to chemotherapy (G: axial CT; H: axial fusion; I: MIP) for metastatic bone lesions (green arrows). PNLD = pelvic lymph node dissection.

The studies were classified as either positive or negative with regard to the identification of findings suggestive of recurrence on the basis of procedure guidelines for PCa imaging (Fig. 1) (*10*). The anatomic sites of the lesions were registered.

PSMA PET/CT findings were compared with histology (when necessary, in the judgment of the clinician); correlative imaging methods, such as CT with contrast, MRI, whole-body MRI, and bone scanning; and clinical and laboratory data (PSA behavior). All data were obtained in the normal care pathway.

Given the composite nature of the standard of reference, we could not calculate sensitivity or specificity; furthermore, a proper evaluation of negative findings was beyond the scope of the present study, which focused on assessing the PSMA PET/CT detection rate (positivity rate), defined as the proportion of patients with positive PSMA PET/CT results.

### Intention to Treat

Before PSMA PET/CT, an intention-to-treat questionnaire was completed by the assistant urooncology teams by the time of referral for evaluation; the treatment categories were radiotherapy only, radiotherapy and antiandrogenic therapy (ADT), salvage lymphadenectomy, ADT only, active surveillance, bilateral orchiectomy, second-generation ADT (abiraterone or enzalutamide), radionuclide therapy, and chemotherapy (taxane).

After the PSMA PET/CT results were made available, the assistant urooncology teams completed the same questionnaire on the basis of the actual treatments used.

### Statistical Analyses

The demographic and clinical variables were tabulated using descriptive analysis. Continuous variables were assessed for the gaussian distribution of the data and presented as mean ± SD, if normally distributed, or median (25th percentile, 75th percentile) if not normally distributed. Comparisons of patients with positive PSMA results and those with negative PSMA results were performed using the *t* test or Wilcoxon–Mann–Whitney test. Discrete variables were presented as proportions and compared between groups using the χ^2^ test. A multivariate logistic regression analysis was performed to identify the independent predictors of positive PSMA results. Variables were selected for the regression model on the basis of significant associations in the univariate analysis and previous clinical knowledge. The level of significance was set at a *P* value of less than 0.05. Analyses were performed using Stata version 15.1 (Stata Corp.).

## RESULTS

### Patient Characteristics

A total of 1,198 PCa patients referred for PSMA PET/CT because of BCR between November 2017 and December 2019 were enrolled; 194 were subsequently excluded because of missing information or loss of follow-up data. Therefore, a cohort of 1,004 patients could be analyzed, here divided by country: Azerbaijan (48), Brazil (165), Colombia (29), India (86), Israel (16), Italy (172), Jordan (26), Lebanon (65), Malaysia (35), Mexico (91), Pakistan (19), Poland (111), South Africa (42), Turkey (57), and Uruguay (*42*). For 2 nations (India and Turkey), data from 2 contributing centers were pooled together for the present study (see the list of participant centers and contributors in the supplemental materials). The distribution of patients according to the Gleason score (GS) was as follows: for a GS of 7, there were 613 patients (61.1%); for a GS of 8, there were 196 patients (19.5%); for a GS of 9, there were 180 patients (17.9%); and for a GS of 10, there were 15 patients (1.5%). The distribution of patients according to PSA levels at PET/CT was as follows: for PSA levels of less than 0.2 ng/mL, there were 41 patients (4.1%); for PSA levels between greater than or equal to 0.2 ng/mL and less than 0.5 ng/mL, there were 188 patients (18.7%); for PSA levels between greater than or equal to 0.5 ng/mL and less than 1 ng/mL, there were 232 patients (23.1%); for PSA levels between greater than or equal to 1 ng/mL and less than 2 ng/mL, there were 235 patients (23.4%); for PSA levels between greater than or equal to 2 ng/mL and less than 4 ng/mL, there were 206 patients (20.5%); and for PSA levels between greater than or equal to 4 ng/mL and less than 10 ng/mL, there were 102 patients (10.2%). The mean PSA doubling time was 11.18 mo (SD, 13.15 mo) (Table 1). Overall, 780 patients (77.7%) were treated initially with radical prostatectomy and 224 (22.3%) were treated with radiotherapy. At the time of the PET scan, the mean time from PCa diagnosis to BCR was 15.6 mo (range, 0.6–43.7 mo); 248 patients (24.7%) were receiving ongoing ADT; and 630 patients (62.7%) had a PSA doubling time of less than or equal to 10 mo.

**TABLE 1:** Patient Characteristics Based on PSMA PET Results

Characteristic	All patients (*n* = 1,004)*	Patients with negative PSMA PET/CT results (*n* = 350)*	Patients with positive PSMA PET/CT results (*n* = 654)*	*P*
Age^†^	67.29 ± 7.48	66.37 ± 7.36	67.77 ± 7.51	0.005
PSA level at time of PET scan				<0.001
<0.2	41 (4.1)	20 (5.7)	21 (3.2)	
0.2–0.5	188 (18.7)	104 (29.7)	84 (12.8)	
0.5–1.0	232 (23.1)	108 (30.9)	124 (19.0)	
1–2	235 (23.4)	77 (22.0)	158 (24.2)	
2–4	206 (20.5)	35 (10.0)	171 (26.1)	
>4	102 (10.2)	6 (1.7)	96 (14.7)	
PSA doubling time^†^	11.18 ± 13.15	12.97 ± 14.04	10.22 ± 12.56	0.002
Initial PSA before therapy^†^	17.27 ± 22.10	14.63 ± 17.69	18.69 ± 24.02	0.006
TNM				<0.001
T1	4 (0.5)	2 (0.6)	2 (0.4)	
T2	439 (56.0)	208 (65.8)	231 (49.4)	
T3	333 (42.5)	103 (32.6)	230 (49.1)	
T4	8 (1.0)	3 (0.9)	5 (1.1)	
Ongoing ADT	248 (24.7)	62 (17.7)	186 (28.4)	<0.001
Radiotherapy as first treatment	224 (22.3)	35 (10.0)	189 (28.9)	<0.001
Time to relapse^‡^	23.0 (8.0, 49.0)	22.5 (8.0, 48.0)	24.0 (9.0, 51.0)	0.57
GS				<0.001
7	613 (61.1)	242 (69.1)	371 (56.7)	
8	196 (19.5)	66 (18.9)	130 (19.9)	
9	180 (17.9)	40 (11.4)	140 (21.4)	
10	15 (1.5)	2 (0.6)	13 (2.0)	
Country income				0.07
High income	390 (38.8)	149 (42.6)	241 (36.9)	
Upper middle income	509 (50.7)	160 (45.7)	349 (53.4)	
Lower middle income	105 (10.5)	41 (11.7)	64 (9.8)	
Continent				0.73
Africa	42 (4.2)	18 (5.1)	24 (3.7)	
Asia	182 (18.1)	64 (18.3)	118 (18.0)	
Europe	388 (38.6)	132 (37.7)	256 (39.1)	
Latin America	392 (39.0)	136 (38.9)	256 (39.1)	

* Data are reported as numbers of patients, with percentages of patients in parentheses, unless otherwise indicated.

^†^Data are reported as mean ± SD.

^‡^Data are reported as median (25th percentile, 75th percentile).

The mean age of patients was 67.3 y (range, 45–87 y); 908 men (90.4%) met the eligibility requirement because they had PSA levels of less than 4 ng/mL, whereas 96 men (9.6%) had PSA concentrations of 4–10 ng/mL and negative MRI, CT, and bone scintigraphy results. The mean PSA level at the time of the PET scan was 1.55 ng/mL. Regarding the stage at presentation, 443 men (44.1%) had clinical stages T1–T2 and 341 (34.0%) had clinical stages T3–T4; in 220 men (21.9%), the T stage was unknown. The mean duration of follow-up after PSMA PET/CT was 16.8 mo (SD, 9.3 mo).

Regarding income, 105, 509, and 390 patients were in the lower middle-income, upper middle-income, and high-income groups, respectively. PSA differences were not significant among them (*P* = 0.94). Of note, there were statistically significant differences regarding PSA doubling time, ongoing ADT, and radiotherapy as the primary treatment among the different income groups. For these 3 groups, the mean PSA doubling times were 9.14, 9.98, and 13.3 mo (*P <* 0.001). There were 40 patients (38.1%), 131 patients (25.7%), and 77 patients (19.7%) receiving ongoing ADT (*P <* 0.001). Radiotherapy was the primary treatment in 42 patients (40.0%), 129 patients (25.3%), and 53 patients (13.6%) (*P <* 0.001).

### PSMA PET/CT

At least 1 malignant lesion was found in 65.1% of the patients (654/1,004), whereas 34.9% (350/1,004) had negative PSMA PET/CT results, with no detectable disease. A summary of the PSMA PET/CT results is shown in Table 1.

**FIGURE 2.**
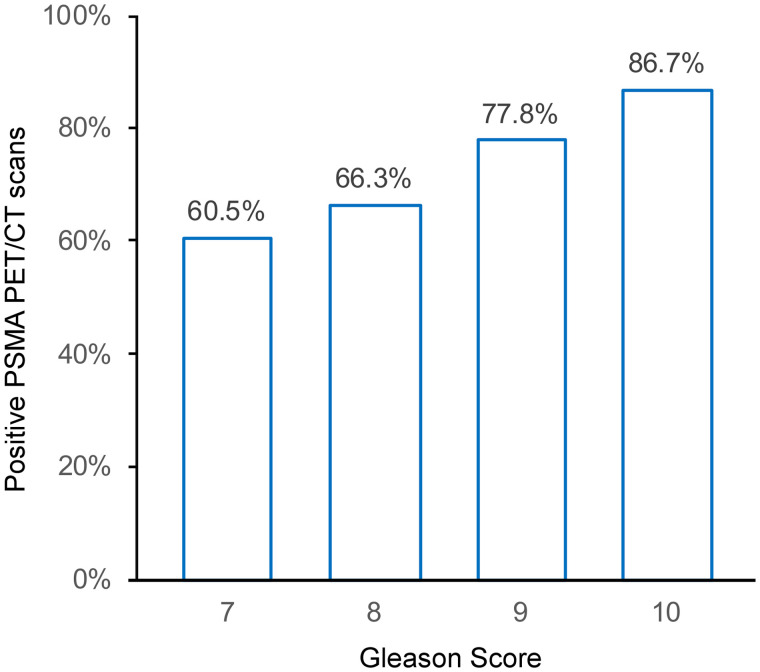
Correlation between PSMA PET/CT positivity and GS.

There was a correlation between PSMA PET/CT and the GS (*P* < 0.001). Detection rates were 60.5% (371/613) for patients with a GS of 7; 66.3% (130/196) for those with a GS of 8; 77.8% (140/180) for those with a GS of 9; and 86.7% (13/15) for those with a GS of 10 (Fig. 2).

**FIGURE 3.**
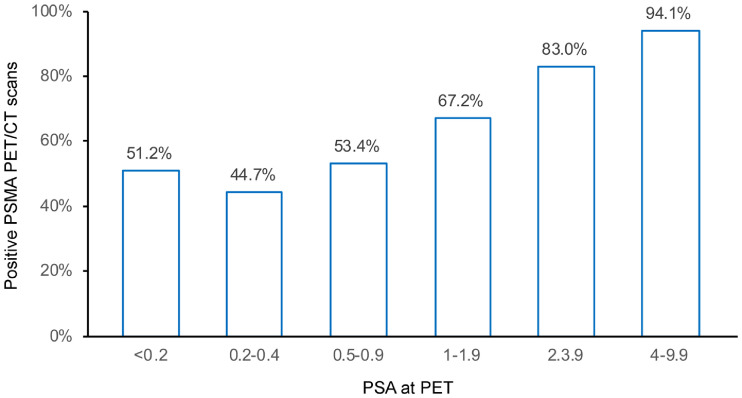
Correlation between PSMA PET/CT positivity and PSA values.

We also found a significant correlation between PSMA PET/CT positivity and PSA values (*P* < 0.001). Detection rates were 51.2% (21/41) for PSA values of less than 0.2; 44.7% (84/188) for PSA values between greater than or equal to 0.2 and less than 0.5; 53.4% (124/232) for PSA values between greater than or equal to 0.5 and less than 1; 67.2% (158/235) for PSA values between greater than or equal to 1 and less than 2; 83.0% (171/206) for PSA values between greater than or equal to 2 and less than 4; and 94.1% (96/102) for PSA values between greater than or equal to 4 and less than 10 (Fig. 3).

**FIGURE 4.**
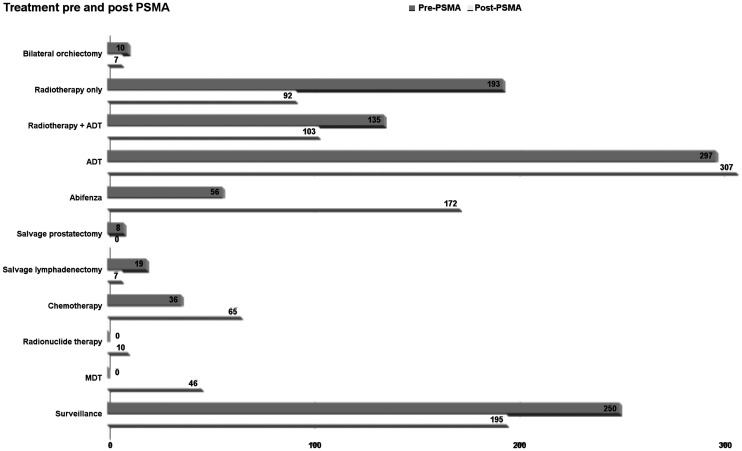
Impact of PSMA PET/CT on clinical management. MDT = metastasis-directed therapy.

PSMA PET/CT results were positive for 69.4% of the patients (437/630) whose PSA doubling times were less than or equal to 10 mo and for 58.0% of the patients (217/374) whose PSA doubling times were greater than 10 mo (*P* = 0.003) (Fig. 4).

The PSMA PET/CT positivity rates per anatomic site were 13.7% (138/1,004) in the prostate or prostatic bed only; 3.9% (39/1,004) in the prostate or prostatic bed and pelvic lymph nodes; 20.5% (206/1,004) in the pelvic lymph nodes only; and 27.0% (271/1,004) in a metastasis at any site (bone only in 10.0% [100/1,004]) (Table 2). In a univariate analysis, factors associated with positive PSMA PET/CT results were age, the PSA level at the time of the PET scan, PSA doubling time, the initial PSA level before therapy, TNM, GS, ongoing ADT, and radiotherapy as the first treatment. Logistic regression showed that PSMA PET/CT positivity was associated with the GS, the PSA level at the time of the PET scan, decreasing PSA doubling time, and radiotherapy as the primary treatment (Table 3).

**TABLE 2:** Positive PSMA PET/CT Studies per Anatomic Site

Anatomic site	Result for positive PSMA PET/CT studies*
Prostate or prostatic bed only	138 (13.7)
Prostate or prostatic bed + lymph nodes	39 (3.9)
Lymph nodes only	206 (20.5)
Metastasis at any site	271 (27.0)
Bone only	100 (10.0)

* Data are reported as numbers of patients, with percentages of patients in parentheses.

**TABLE 3.** Association of Clinical Covariates with Likelihood of Detection by PSMA PET/CT

Covariate	Odds ratio	*z*	*P*	95% CI	
				Lower	Upper
Age	1.01	1.69	0.091	0.99	1.03
PSA level at PCa diagnosis	0.99	−0.05	0.958	0.99	1.01
GS	1.37	3.30	0.001	1.25	1.65
at time of PSMA PET/CT	1.72	7.57	0.001	1.47	1.97
PSA doubling time	0.98	−3.30	0.001	0.97	0.99
Ongoing ADT	1.23	1.14	0.255	0.93	1.76
Radiotherapy first	2.17	3.56	0.001	1.42	3.34

Of the 1,004 cases included in the present study, 12.4% (124 patients) had doubtful PET findings (as reported by local readers). Of these, 90 patients had other positive findings, regardless of the indeterminate one(s); thus, their scans were already defined as positive PSMA PET/CT scans. Of the remaining 34 patients (3.4%) in whom the indeterminate lesion at PSMA PET/CT was the sole finding, 3 were confirmed to have true-positive results on the basis of follow-up data, whereas 31 (3.1%) were regarded as having false-positive results (encompassing reactive lymph nodes, bone fractures, trauma, and benign pulmonary lesions).

### Impact of PSMA PET/CT on Clinical Management

Disease management changed in 56.8% of our cohort (570/1,004) after PSMA PET/CT information was obtained. The following changes occurred as a result of PSMA PET/CT: 77 patients underwent active surveillance, 35 underwent radiotherapy only, 55 underwent radiotherapy and ADT, 152 underwent ADT only, 48 underwent salvage lymphadenectomy, 5 underwent bilateral orchiectomy, 140 underwent second-generation ADT (abiraterone or enzalutamide), 10 underwent radionuclide therapy, and 48 (patients with polymetastatic disease) started taxane chemotherapy.

Of the patients for whom there was no management change motivated by PSMA PET/CT results (434/1,004; 43.2%), 118 remained under active surveillance, 57 underwent radiotherapy only, 48 underwent radiotherapy and ADT, 5 underwent salvage lymphadenectomy, 155 underwent ADT, 2 underwent bilateral orchiectomy, 32 underwent second-generation ADT (abiraterone or enzalutamide), and 17 (patients with polymetastatic disease) started taxane chemotherapy (Fig. 4).

### PSMA PET/CT Worldwide

The centers were grouped in 2 distinct ways: by country income (high income: Israel, Italy, Poland, and Uruguay; upper middle income: Azerbaijan, Brazil, Colombia, Jordan, Lebanon, Malaysia, Mexico, South Africa, and Turkey; and lower middle income: India and Pakistan) and by continent (Africa, America, Asia, and Europe). There were no significant differences in PSMA PET/CT positivity by lower middle income, upper middle income, and high income (61%, 69%, and 62%, respectively) or by continent (Africa: 57%; Asia: 65%; Europe: 66%; and Latin America: 65%) (*P =* 0.07 and *P =* 0.73, respectively) (Table 1).

## DISCUSSION

Our findings resonate with the available literature on the use of PSMA PET/CT in the evaluation of PCa patients in the scenario of BCR (*3*–*8*,*10*,*20*–*44*). We analyzed 4 main aspects of PSMA PET/CT in this setting: positivity rate, clinical factors associated with PSMA positivity, differences in performance with regard to continents and incomes, and impact on clinical management. The PSMA PET/CT positivity rate was 65.1%, similar to the positivity rates reported in other studies, ranging overall from 63% to 75% (*10*,*14*,*16*,*21*,*22*). Also, increasing PSA levels at the time of the scan were associated with higher PSMA PET/CT positivity, with rates similar to those previously reported (Supplemental Table 1); the exception was higher PSMA PET/CT positivity in the group with PSA levels of less than 0.2 ng/mL compared with the mean in the available literature: 51.2% versus 36.8% (*3*–*8*,*10*,*20*–*44*). This difference might be explained by the small number of patients in this group in our cohort (*41*) but also by the small number of patients evaluated in the cohort of all patients (316). Nevertheless, 51.2% falls into the range observed in the literature (11.3%–58.3%). In the other scenarios (PSA levels of <0.5, <1.0, and <2.0 ng/mL), the positivity rates were quite similar (44.7% vs. 43.3%; 53.4% vs. 52.2%, and 67.2% vs. 58.9%, respectively).

The observed location of malignant lesions is in agreement with those in previous reports, with lymph nodes being the principal site of recurrence (24.4%), followed by local recurrence in the prostate bed (17.6%), and with any metastatic disease in 27.0% (*9*,*45*).

Furthermore, higher positivity rates were also associated with features of advanced or aggressive disease other than increasing PSA levels: a shorter PSA doubling time (≤10 mo) and a higher GS. These findings are also in line with the current available literature (*42*,*46*) and are likely due to the presence of more neoplastic lesions and to higher tumoral cell turnover, which provide more available sites for PSMA ligand binding and, thus, lead to positive PET/CT results.

One interesting finding was the association of radiotherapy as a primary radical treatment with PSMA PET/CT positivity in the BCR setting. Although patients receiving radiotherapy represented only 22.3% of all patients, they comprised 28.9% of patients with positive PSMA PET/CT results (*P* < 0.001). It is already known that, in comparison to radical prostatectomy, radiotherapy is associated with higher BCR rates (*46*). Our results suggest that in addition to having more frequent residual/recurrent disease, these patients are also more likely to have positive PSMA PET/CT scans in the BCR setting.

The most relevant finding is that there were no statistically significant differences in PSMA PET/CT performance among continents or among the different income categories in which the participants were distributed. This finding is important because it highlights the fact that the great heterogeneities among nations do not seem to interfere with each country’s capacity to provide high-quality PSMA PET/CT studies in the appropriate medical centers.

PSMA PET/CT affected clinical management in more than half of our cohort, as the therapeutic strategy was altered by PSMA PET/CT results 56.8% of the time, similar to previous reports in different studies (*13*,*16*,*21*).

Regarding the limitations of the present study, a major one is that histopathology as a gold standard was available only in a few cases. It is well known that histopathologic confirmation in all patients is not feasible because of practical and ethical issues. Hence, in most patients, a composite standard of reference (histopathology and clinical and laboratory evaluations) was used. Another important limitation is the relatively small percentage of patients included in low-income countries and in Africa. Furthermore, South Africa’s income and PSMA PET/CT availability are not representative of the continent. Moreover, regarding the impact of PSMA PET/CT on clinical management, the available data unfortunately do not permit an evaluation of its effects on survival rates.

The endeavor of performing this multicenter, international study, enrolling more than 1,000 patients from many countries was made possible only through the combined efforts of several different researchers and the support of the International Atomic Energy Agency, a nonprofit agency, which enabled gathering of this large and diverse cohort.

## CONCLUSION

This multicenter, international, prospective trial of PSMA PET/CT confirms its capability for detecting local and metastatic recurrences in most PCa patients in the setting of BCR. PSMA PET/CT positivity was correlated with the GS, the PSA level at the time of the PET scan, PSA doubling time, and radiotherapy as the primary treatment. PSMA PET/CT results led to changes in therapeutic management in more than half of the cohort. The present study demonstrates the reliability and feasibility of PSMA PET/CT in the workup of PCa patients with BCR.

## DISCLOSURE

This research was partially funded by IAEA. No personal grants, consulting fees, or honoraria were involved in the present work. No other potential conflict of interest relevant to this article was reported.

## Notes

KEY POINTS **QUESTION:** In a large international cohort of PCa patients in the setting of BCR, how similar are PSMA PET/CT positivity rates and impact on clinical management among countries on different continents and with different incomes? **PERTINENT FINDINGS:** PSMA PET/CT positivity was correlated with the GS, serum PSA levels, and radiotherapy as the primary treatment. An impact of PSMA PET/CT results on clinical management was observed in most cases, and all findings were similarly consistent regardless of the country. **IMPLICATIONS FOR PATIENT CARE:** Our results confirm the worldwide feasibility and usefulness of PSMA PET/CT in the setting of PCa BCR.
